# Mapping of the characteristics in the CoDAS journal publications in the voice area: a scoping review

**DOI:** 10.1590/2317-1782/20232022088en

**Published:** 2023-06-05

**Authors:** Walter Williams Albrechet Chamun, Vanessa Veis Ribeiro, Glaucya Madazio, Mara Behlau

**Affiliations:** 1 Centro de Estudos da Voz - CEV - São Paulo (SP), Brasil.; 2 Departamento de Fonoaudiologia, Universidade Federal da Paraíba - UFPB - João Pessoa (PB), Brasil.; 3 Departamento de Fonoaudiologia, Universidade Federal de São Paulo - Unifesp - São Paulo (SP), Brasil.

**Keywords:** Voice, Outcomes, Procedures, Dysphonia, Voice Disorders

## Abstract

**Purpose:**

To map and describe the characteristics present in the publications of the CoDAS journal in the voice segment.

**Research strategies:**

The research was carried on the Scielo database using the descriptor voice.

**Selection criteria:**

CoDAS publications in the field of voice.

**Data analysis:**

Specific data collected according to delineation, summarized by descriptive analysis and analyzed in narrative format.

**Results:**

Studies published in 2019 and with cross-sectional delineation were more frequent. The most frequent result in the cross-sectional studies was the vocal self-assessment. Most intervention studies were of immediate single-session-only effect. The most frequent procedures in the validation studies were translation and transcultural adaptation.

**Conclusion:**

There was a gradual increase in the number of publications of voice studies, though these had heterogeneous characteristics.

## INTRODUCTION

In the contemporary healthcare world, the search for a scientific knowledge in the contemporary world of health care, the search for scientific knowledge has taken a prominent place, with the main purpose of fostering clinical practice. In this sense, the production of evidence through the publication of a scientific article is a way to transmit to the scientific and clinical community data on the development of new procedures, instruments, and interventions, as well as the analysis of risk factors and epidemiological reality in the various areas of Science^(1),^ such as in Speech Therapy (phonoaudiology).

In Brazil, voice is one of the phonoaudiological areas recognized as a specialty since 2006^(2)^. Since this resolution, the voice area has advanced even more as a recognized specialty in constant evolution. The strength and recognition of a profession or area are in most part based on the quality of the articles published in peer-reviewed journals. The Brazilian scientific production in the area of voice is vast, which contributes to and strengthens the practice. Historically, the first researches in the voice area, as well as in the health and speech sciences in general, dealt with the delineation of expert opinions and case series from clinical practice. These were the first scientific steps in a young field, but the conclusions of these studies could not yet be generalized. The practice was based on the learning acquired during the clinicians' training, and their questions were answered directly with recognized experts. Over time, research has been improving, and the focus has become the availability of evidence to improve clinical practice, as well as the implementation of evidence. Currently, the challenge for vocal clinicians is to choose the best scientific evidence to support their practice^(3,4).^ These changes stemmed, in large part, from a movement called Evidence Based Practice (EBP), which seeks to ensure the quality of care of health professionals, including speech therapists, aiming at better decision making for each case^(5).^ The EBP consists of three practices: 1) The clinician identifies a problem or doubt in their practice; 2) The researcher transforms this problem/doubt into a research question and develops a study to answer it based on evidence; 3) The clinician searches and selects the study, and applies the evidence to their clinical practice, from a critical analysis that takes into account their practice experience and patient perspectives^(6).^ Thus, the execution of EBP does not depend only on researchers, but mainly on clinicians, who must act as integrators of science and clinic, critical evaluators of data, and executors in the application of evidence in their clinical cases^(7).^ It can be said that the continuous development of Speech Pathology and the field of voice is directly linked to the practice of EBP^(8),^ although EBP is still not frequent in the field of voice.

Brazil currently hás four speech therapy specialized magazines that contribute to EPB, one of them is CoDAS (*Communication Disorders, Audiology and Swallowing*) an easy and short english abbreviation that includes the main areas of Speech Therapy. CoDAS is a journal that has been adapted to the needs of the phonoaudiological academy and clinic. Initially, it was called Pró-Fono Revista de Atualização Cientifica (2005-2010). In 2010, it was renamed Jornal da Sociedade Brasileira de Fonoaudiologia (2010-2012). The articles published from 2010 onwards are available on the Scielo platform with open access. In its last restructuring in 2013, it was renamed CoDAS, with a centralized editorial as the only publication of the Brazilian Society of Speech Therapy. The journal is indexed by *Web of Science*, MEDLINE/PubMed, Scopus, PsycINFO, *Scientific Electronic Library Online* (SciELO), *Linguistics and Language Behavior Abstracts* (CSA), Literatura Latino-Americana e do Caribe em Ciências da Saúde (LILACS), *SociedadIberoamericana de Información Científica* (SIIC Data Bases), and the *Directory of Open Access Journals* (DOAJ). Considering the importance of this journal for research and clinical practice in the area of voice, it is important to map the publications in this area that have been published in it. Such data will provide a general panorama (landscape) of the scope of the publications in regards to the designs, samples, outcomes and exercise prescription. It is believed that such data will contribute to identify what has been done in the country, map the advances, point out the limitations and needs of the area and help the speech therapist in the search for evidence towards a final goal that is to provide the best care for patients in the vocal clinic. Furthermore, the mapping done by a scope review can contribute to future directions in the area and the growth of science.

Thus, the overall objective of this research was to map and describe the characteristics of CoDAS journal publications in the area of voice.

## RESEARCH STRATEGY

The present study has a scoping review design and followed the recommendations of *Joanna Briggs Institute Manual for Evidence Synthesis for Scoping Reviews* ^(9)^ and PRISMA-ScR^(10)^. The protocol of the present scope review was registered at Open Science Framework (doi:10.17605/OSF.IO/VFWN7). To elaborate the research question we used the acronym PCC: Population - dysphonic and non-dysphonic individuals; Concept - sampling and methodological characteristics; Context - CoDAS magazine. Thus, the research question that supported its development was: What are the main sampling and methodological characteristics of studies with dysphonic and non-dysphonic individuals published in CoDAS?

The search was conducted in the Scielo database, using the descriptor “voice”. Journal filters were applied, selecting only CoDAS, and publication period until December 2019. The search was performed in the month of August 2020.

The inclusion criteria used to consider the studies for this review were: articles published in the journal CoDAS (Pró-Fono Revista de Atualização Científica, Jornal da Sociedade Brasileira de Fonoaudiologia e CoDAS), period until December 2019, in the area of voice, with a population of dysphonic and non-dysphonic individuals. In June 2022 the article was updated with publications from January 2020 to December 2021. We excluded interdisciplinary studies in which the focus was not the area of voice (outcomes of other areas), and secondary studies (literature review).

The procedures used to select the studies and apply the eligibility criteria were: reading the title; reading the abstract, and reading the full articles. The selection was performed by the main author between August and November 2020.

## ANALYZING DATA

Data extraction and analysis was performed by two authors. In order to facilitate data extraction and analysis, the selected studies were separated into three groups, according to the design: observational^(11);^ intervention^(11);^ and instrument validation. The data extracted from the studies were:

1. Observational (Cross-sectional, Cohort and Case-control): authors, year, country, institution, design, area, age group, gender, sample size, outcomes, self-assessment, perceptual-auditory assessment, acoustic analysis, aerodynamics.
2. Intervention (Quasi-experimental, Experimental and Intervention before and after): authors, year, country, institution, design, area, age group, gender, sample size, number of sessions, session time, session frequency.
3. Translation, cross-cultural adaptation, and validation of instruments: authors, year, country, institution, design, area, age group, gender, sample size, translation, and cross-cultural adaptation.

The synthesis of the data was presented descriptively by means of tables and graphs with frequency analysis. Data analysis was performed in narrative format.

## RESULTS

**Figure 1:**
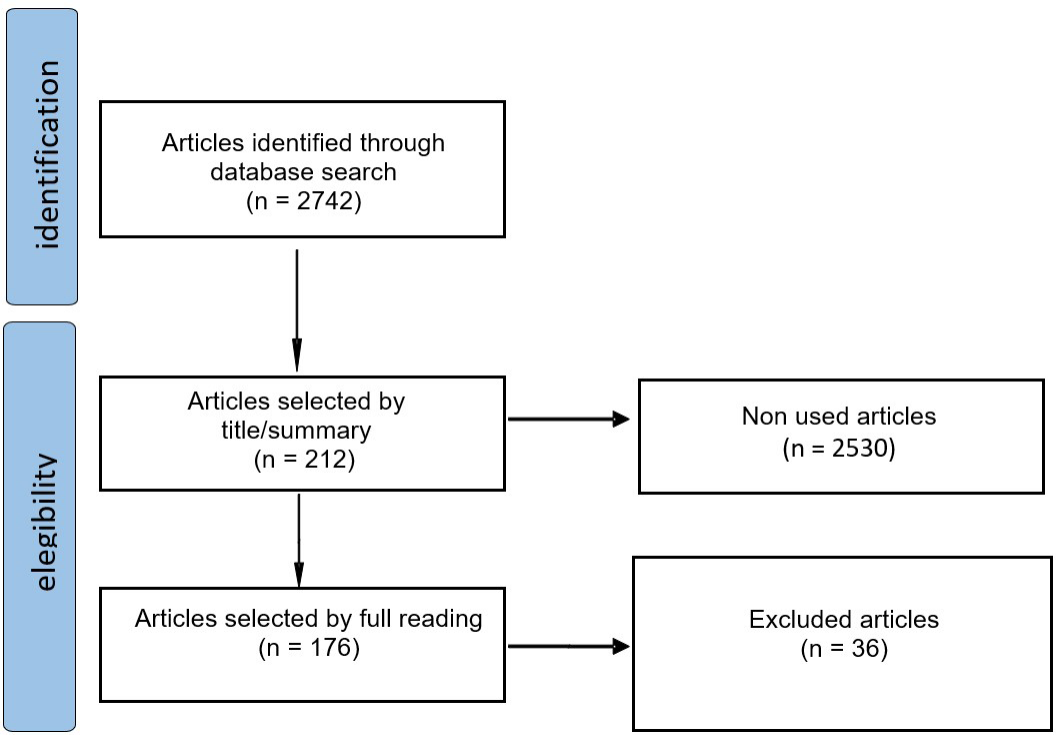
Search and selection flowchart

Figure 1 shows that 2742 studies were identified in the Scielo database. During the first selection phase 2530 studies were excluded because they were not in the area of voice or because they were interdisciplinary studies with no voice endpoints, thus making 212 studies. During the second selection phase, 36 were excluded based on the design (secondary studies). Thus, 176 studies were selected^(12-187).^

n = Absolute frequency

**Figure 2:**
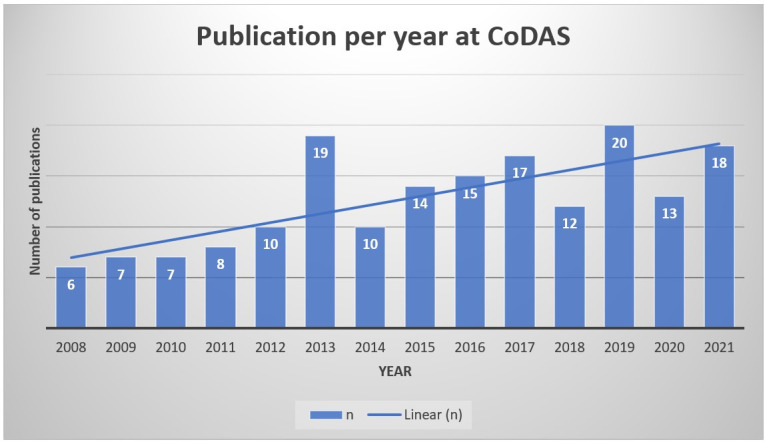
Descriptive analysis of the frequency of voice articles per year of publication in CoDAS

Publications in the area of voice were most frequent in the years 2019 (n=20; 11.4%) and 2013 (n=19; 10.8%), as indicated in Figure 2.

Table 1 shows that in CoDAS the articles developed in Brazil were more frequent (n=165; 93.8%), followed by the United States of America (n=3; 1.7%). The institutions that published the most were Centro de Estudos da Voz (n=46; 26.1%), Universidade de São Paulo (n=20; 11.4%), and Universidade Federal de São Paulo (n=17; 9.7%), tied with multicenter studies (n=17; 9.7%).

**Table 1:** Studies characteristics

Variables and categories	n	%
**Country**		
Brazil	165	93.8
Chile	3	1.7
United States	3	1.7
Belgium	1	0.6
Brazil & Belgium	1	0.6
Brazil & Canada	1	0.6
Netherlands	1	0.6
Italy	1	0.6
**Institution**		
Centro de Estudos da Voz - São Paulo / SP	46	26.1
Universidade de São Paulo - São Paulo / SP	20	11.4
Multicêntricas	17	9.7
Universidade Federal de Minas Gerais - Belo Horizonte / MG	17	9.7
Universidade Federal de São Paulo - São Paulo / SP	16	9.1
Universidade Federal da Paraíba - João Pessoa / PB	10	5.7
Pontifícia Universidade Católica de São Paulo - São Paulo / SP	7	4.0
Universidade Estadual Paulista - Marília / SP	5	2.8
Universidade Federal de Santa Catarina - Florianópolis / SC	4	2.3
Universidade Federal do Espírito Santo - Vitória / ES	4	2.3
Universidade Federal de Santa Maria / RS	3	1.7
Santa Casa de Misericórdia de São Paulo - São Paulo / SP	3	1.7
Universidade Metodista de Piracicaba - Piracicaba / SP	3	1.7
Universidade Estadual do Centro-Oeste-Irati / PR	2	1.1
Universidade Federal da Sergipe - Lagarto / SE	2	1.1
Columbia University - New York (USA)	1	0.6
Hospital AG Sint-Jan - Bruges (Belgium)	1	0.6
Pontificia Universidade Católica de Goiás - Goiania / GO	1	0.6
Santa Casa de Misericórdia de Porto Alegre - ISCMPA - Porto Alegre / RS	1	0.6
Santa Casa de Misericórdia do Rio de Janeiro - Rio de Janeiro / RJ	1	0.6
Southern IIIinois University - Carbondale / IL (USA)	1	0.6
The Touro College and University System - New York (USA)	1	0.6
Universidad de Valparaiso - Valparaiso / Chile	1	0.6
Universidade Estadual de Campinas - Campinas / SP	1	0.6
Universidade Estadual de Montes Claros - Montes Claros/MG	1	0.6
Universidade Federal da Bahia - Salvador / BA	1	0.6
Universidade Federal de Ciências da Saúde de Porto Alegre - Porto Alegre / RS	1	0.6
Universidade Federal de Pernambuco - Recife/PE	1	0.6
Universidade Federal do Rio Grande do Sul - Porto Alegre / RS	1	0.6
Universidade Tuiuti do Paraná-UTP-Curitiba / PR	1	0.6
Universidade Veiga de Almeida e Ambulatório de Urologia Reconstrutura Universitário Pedro Ernesto - HUPE - Rio de Janeiro / RJ	1	0.6
Zuyd University of Applied Sciences - Heerlen (Holanda)	1	0.6
**Outline**		
Observational-Cross-sectional	115	65.3
Quasi-experimental	27	15.3
Translation. Cross-cultural adaptation and Validation of instruments	24	13.6
Experimental	6	3.4
Intervention before and after	4	2.3

Caption: n = Absolute frequency; % = percentage relative frequency

Most studies were cross-sectional (n=115; 59.7%), followed by quasi-experimental (n=27; 15.3%), translation, cross-cultural adaptation or validation studies (n=24; 13.6%), experimental (n=6; 3.4%) and before-and-after intervention (n=4; 2.3%).

According to Table 2, there was a higher frequency of clinical voice studies (n=105; 59.7%). There were more frequent studies with sample in the age range of 18-59 years (n=96; 54.5%), and of both genders (n=102; 58.0%).

**Table 2:** Characteristics of the area and sample of the studies

Variables and categories	n	%
**Area**		
Clinical Voice	105	59.7
Professional Voice	71	40.3
**Age range**		
18-59	96	54.5
60-103	42	23.9
0-18	21	11.9
Non informed	17	9.7
**Gender**		
Both	102	58.04
Feminine	48	26.57
Non informed	17	9.09
Masculine	9	6.29

Caption: n = Absolute frequency; % = percentage relative frequency

Regarding the 115 cross-sectional studies, Table 3 shows that the most frequent outcomes were vocal self-assessment (n=102; 88.7%), the perceptual-auditory voice judgment - JPA (n=92; 80%) and the acoustic voice analysis (n=61; 53%).

**Table 3:** Characteristics of cross-sectional studies (n=115)

Variables and categories	n	%
**Outcomes (n=115)**		
Self-evaluation	102	88.7
JPA	92	80.0
Acoustic analysis	61	53.0
Evaluations of other areas	42	36.5
Laryngeal image	22	19.1
Aerodinamics	20	17.4
Other vocal evaluations	10	8.7
**Self-evaluation (n=102)**		
ESV	14	13.7
QVV	10	9.8
IDV-10	7	6.9
Self-assessment elaborated by the researcher	6	5.9
IDV	6	5.9
IFV	6	5.9
CPV-P	5	4.9
EDTV	5	4.9
ITDV	5	4.9
QSHV	5	4.9
PPAV	4	3.9
URICA-VOZ	4	3.9
IDCM	4	3.9
PRRD	3	2.9
LSSV	2	2.0
SSPS	2	2.0
VcD	2	2.0
BORG CR10-BR	1	1.0
EAFV	1	1.0
IDCC	1	1.0
IDV-C	1	1.0
List of vocal signs and symptoms	1	1.0
LSU-BR	1	1.0
PDA	1	1.0
PEED	1	1.0
Vocal quality and intensity	1	1.0
QVV-P	1	1.0
RAVI	1	1.0
TVQ	1	1.0
**JPA (n=92)**		
Scales created by researchers	26	28.3
GRBASI	28	30.4
EAV- visual analog scale	21	22.8
CAPE-V	12	13.0
EN (Numerical Scale)	1	1.1
GGDV (overall degree of voice deviation)	1	1.1
GT (degree of tension)	1	1.1
VPAS	1	1.1
Likert (scale)	1	1.1
**Acoustic analysis (n=61)**		
Traditional acoustic analysis	52	85.2
DDF (phonatory deviation diagram)	5	8.2
DDC (diadocokinesis)	2	3.3
AVQI	1	1.6
CPPs	1	1.6
**Aerodynamics (n=20)**		
TMF (maximum phonatory time)	19	95.0
Nasometry	1	5.0

Caption: n = Absolute frequency; % = percentage relative frequency; JPA = Auditory Perceptual Judgment; ESV = Vocal Symptomscale QVV = Quality of Life in Voice; IDV-10 = Vocal Handicap Index; IDV = Vocal Handicap Index; IFV = Vocal Fatigue Index; CVP-P = Teacher's Vocal Production Condition; EDTV = Vocal Tract Discomfort Scale; ITDV = Hearing Index for Voice Disorder; QSHV = Health and Vocal Hygiene Questionnaire; PPAV = Participation Profile Protocol and Vocal Activities; IDCM = Modern Corner Handicap Index PRRD = Dysphonia Risk Screening Protocol; LSSV = List of Vocal Signs and Symptoms; SSPS = Scale for Self-Assessment when Speaking in Public; VcD = Questionnaire Living with Dysarthria; BORG CR10-BR = *Adapted Borg CR10 For Vocal Effort Ratings*; EAFV = Vocal Fatigue Self-Perception Scale; IDCC = Vocal Handicap Index in Classical Singing; IDVC = Corner Handicap Index; LSU-BR = *The Levels of Speech Usage*; PDA = Autonomous Dysfunction Protocol; PEED = Dysphonia Coping Strategy Protocol; QVV-P = Quality of Life in Pediatric Voice; RAVI = Vocal Alteration Screening in the Elderly; TVQ = *Transgender Voice Questionnaire for Male to Female Transsexuals*; GRBASI = Japanese scale for evaluation of auditory perceptual parameters; EAV = Visual Analog Scale; CAPE-V = Consensus on auditory auditory perceptual evaluation of voice; EN = Numerical Scale; GGDV = Global Degree of Voice Deviation; GT = Degree of Tension; VPAS = John Laver's Vocal Profile Analysis Protocol; LIKERT = Self-report scale; DDF = Phonatory Deviation Diagram; DDC = Diadocokinesia Review; AVQI = Acoustic Voice Quality Index; CPPS = *Smoothed Cepstral Peak Prominence*; TMF = Maximum Phonatory Times; Nasometria = Nasallance Assessment

In studies which analyzed the self-reported impact of a voice problem, that is, voice self-assessment (n=102), the most used instruments were the Vocal Symptoms Scale - VSS (n=14; 13,7%), Quality of Life in Voice - QLV (n=10; 9,8%) and the Vocal Disadvantage Index - VDI-10 (n=7; 6.9%). In the studies that performed JPA (n=78), the GRBASI scale (n=28; 30.4%) and self-generated scales (n=26; 28.3%) were most frequently used. In the 61 studies with acoustic analysis, traditional acoustic parameter extraction (n=52; 85.2%) and the phonation deviation diagram (POD) (n=5; 8.2%) were the most published. Finally, the few papers which did aerodynamic analysis (n=20) studied maximum phonatory time - TMF (n=19; 95%) and nasometry (n=1; 5%).

It was observed that of the 37 intervention studies, 16 (43.2%) studied immediate effects, followed by those that did six (n=3; 8.1%) or twelve intervention sessions (n=3; 8.1%).

The most frequent session time among those who reported the information was 60 minutes (n=6; 16.2%), and the absence of this information was predominant (n=10; 27%). Studies with a single session frequency (n=16; 43.2%) were the most frequent, followed by weekly frequency (n=9; 24.3%), as shown in Table 4.

**Table 4:** Characteristics of intervention studies (n=37)

VARIABLES AND CATEGORIES	n	%
**Number of sessions**		
1 (immediate effect)	16	43.2
6	3	8.1
12	3	8.1
2	2	5.4
5	2	5.4
16	2	5.4
3	1	2.7
4	1	2.7
7	1	2.7
8	1	2.7
10	1	2.7
45	1	2.7
Does not apply	3	8.1
**Session time**		
Non informed	10	27.0
60 minutes	6	16.2
20 minutes	3	8.1
1 minute	3	8.1
Does not apply	3	8.1
30 minutes	2	5.4
3 minutes	1	2.7
5 minutes	1	2.7
10 minutes	1	2.7
15 minutes	1	2.7
16 minutes	1	2.7
40 minutes	1	2.7
45 minutes	1	2.7
64 minutes	1	2.7
90 minutes	1	2.7
1 session of 240 minutes / 4 sessions of 120 minutes	1	2.7
**Session Frequency**		
Once (immediate effect)	16	43.2
Once a week	9	24.3
Twice a week	3	8.1
Non informed	3	8.1
2 to 3 times a week	1	2.7
4 times a week	1	2.7
Once a month	1	2.7
Does not apply	3	8.1

Caption: n = Absolute frequency; % = percentage relative frequency

Table 5 shows that of the 24 elaboration, adaptation, and validation studies, 17 (70.8%) performed the translation and cross-cultural adaptation phase; 5 (20.8%) performed validation; and, only 2 (8.3%) elaborated a new instrument.

**Table 5:** Characteristics of translation studies, cross-cultural adaptation and validation (n=24)

Variables and categories	n	%
Translation and cross-cultural adaptation	17	70.8
Validation	5	20.8
Instrument development	2	8.3

Caption: n = Absolute frequency; % = percentage relative frequency

## DISCUSSION

Scientific research in Speech Therapy seeks to provide reliable evidence for the clinic^(159,163,185-187)^ based on evidence-based practice^(188).^ In this practice, clinicians are faced with different results, and need to compare evidence that answers their questions and assists in decision making in search of the best results. However, this comparison is made difficult when analyzing research with different procedures or with several instruments used for the same purpose. The CoDAS journal occupies a prominent place in the national scenario of scientific research publications. In this sense, mapping procedures and analyzing their frequencies show trends, limitations, and can contribute to clinical practice, as well as to the development of other studies.

The results of the present scoping review showed that there was a non-linear upward curve of publications in the CoDAS journal in the last years analyzed, with a rise in the years 2013 and 2020. The higher number of publications in the year 2013 can be explained by changes in editorial strategies, such as the new name of the journal that began to be called CoDAS, the insertion of area editors, the greater participation of foreign colleagues and the professionalization of the editorial office. In addition, the year 2013 presented an improvement in the definition of the objectives of Brazilian studies in the designs of experiments, as well as the expansion of multicenter papers^(189).^ As for the year 2019, the journal was marked by improved indexing and internationalization of the journal.

National publications are the most frequent in CoDAS. Among the 11 international articles, the United States of America and Chile have published three, but it is worth remembering that only recently the journal has had international visibility with English as the mandatory language. The international articles are published both jointly between domestic and foreign institutions, and with only foreign authorship. It is believed that, even in the face of a journal that is still strong and recognized in Brazilian Speech Therapy publications, the increase in internationalization, such as the recent indexation of the journal in the Web of Science, will bring greater international visibility and reach, besides more growth and research data for the journal.

The Centro de Estudos da Voz - CEV was the educational institution that published the most in CoDAS in the area of voice, followed by Universidade de Sao Paulo - USP, Universidade Federal de Sao Paulo - UNIFESP and the multicenter publications. CEV is a teaching and research institution in the area of human communication that completed 40 years in 2021, that offers a Specialization Course in Voice - CECEV, whose first graduating class was in 1993 (The teacher's voice, Sinpro/CEV). In 2021, CECEV had its 24th class, and all the students are encouraged and oriented to develop a monograph to conclude the course, to be presented in congresses and published. USP is a traditional public university in the area, which has three campuses with the Speech Therapy course, in São Paulo, Ribeirão Preto, and Bauru. UNIFESP is one of the oldest universities in teaching and research, and is also recognized for its scientific effort in the area of voice. It is a public university that aims at developing interrelated teaching, research and extension activities, with emphasis on the specific field of health sciences^(190).^ The UNIFESP counts on scientific initiation programs while still in the undergraduate course, and the improvement and specialization programs are linked to scientific production and publication. In the sequence, the results pointed to multicenter publications. This is a worldwide trend and a good indication for the journal, showing that researchers from institutes and universities have been coming together for the improvement of scientific development. The expansion of multicentric works can offer a more comprehensive national geographical representation, besides being fundamental for the development of large projects.

Observational studies of the cross-sectional type, which are simple, fast, and easier to execute designs, were most commonly found in the review. They are research designs that are thought to be an excellent method for describing characteristics of a population. However, they are valid only for that particular place and time, and over time their findings can no longer be used for the clinic^(191,192).^ It is important to highlight that the design is directly related to the clinical question that is intended to be answered, and this type of study is more indicated for the description of characteristics, diagnostic accuracy and disease prevalence^(3).^ Cross-sectional studies were followed by quasi-experimental studies, the most frequent type among intervention studies. Quasi-experimental studies are controlled interventions without sample randomization. The lack of randomization generates a risk of selection bias. Experimental studies, also known as randomized clinical trials, are considered high level of evidence studies^(3)^ and the gold standard for efficacy analysis of interventions^(192),^ but only six publications with this design were observed. Although the quality of publications has increased in the last decade, being still in the process of growth and structural modifications, few researches published in specialized journals incorporated the proper methodology and answered the clinical questions satisfactorily, both in Brazilian journals and in international publications^(193)^.

A balance was observed between studies of clinical voice and professional voice, however, still with greater frequency in the area of clinical voice, historically more present in the area of Voice. It is important to note that both subareas have been contemplated in CoDAS’ publications journal, expanding the possibilities of searching for evidence for clinical vocal practice, professional voice training, and clinical voice therapy.

Most studies included adult participants (18-59 years), followed by the elderly (60-103 years) and children and adolescents (0-18 years). Adults also represent the majority of patients who seek vocal clinic^(194).^ The elderly population has been gaining space not only in vocal clinic, but in all studies in the health area, by demographic changes and the search for vocal longevity and quality of life, although they are still few compared to adults^(195,196)^.

Children are the minority in seeking clinical care, although the prevalence data of childhood dysphonia is up to 38% of the pediatric population^(151)^. It is believed that pediatric dysphonia has fewer studies by the need for parental consent that do not necessarily want their children as a research subject^(151)^. The occurrence of studies with children found in this review is in line with the literature^(151,196)^. It is noteworthy here that some research did not report the age range of participants, although it is essential data for the applicability and interpretation of results.

Most studies included both genders in their analyses. This data is positive and relevant because the anatomical and physiological differences between the sexes lead to the need for specific studies and different interpretations, besides the different normality values for some evaluations^(197-199)^.

When considering only the cross-sectional studies, it is observed that the most frequent outcome was self-assessment, followed by JPA. These two assessments are part of the non-instrumental assessments that make up the multidimensional evaluation of voice^(6)^. Self-evaluation brings data that cannot be obtained in the clinical evaluation carried out by the speech therapist and is used to quantify the perception of the subject about the influence of his or her voice on different daily activities^(200)^. Total wellbeing does not include only the absence of disease, but also the individual's self-perception of his condition and the impacts on several aspects of his life^(201-203)^.

The review showed that the most frequently used self-assessment instruments were the the Vocal Symptoms Scale - VSS, Quality of Life in Voice - QLV and the Vocal Disadvantage Index - VDI-10.

These instruments, all validated for Brazilian Portuguese^(23,37,204)^ are widely used in day to day vocal clinic for being fast, easy to apply and also for presenting reliable psychometric properties, capable of classifying individuals with and without dysphonia. The VSS is one of the most robust instruments in the area of voice because of its psychometric properties and seeks to analyze the self-perception of vocal symptoms^(37)^. The IDV-10, a reduced version of the IDV-30, is a quick, practical and reliable instrument to measure vocal handicap in individuals with voice problems^(68)^. The QLV was the first instrument of vocal self-assessment to be validated for Brazilian Portuguese, widely applied in vocal clinic, which allows the analysis of the impact of a voice problem in the quality of life of dysphonic individuals^(204)^.

When caring for a dysphonic patient, the *American Speech-Language-Hearing Association* (ASHA) recommends the use of self-assessment protocols, but does not cite a specific one^(6)^ probably because the psychometric properties of the instruments are different between language validations and because the instruments have different objectives.

The JPA is considered the gold standard in vocal evaluation, capable of qualifying voice quality and quantifying the degree of deviation^(205)^. Most cross-sectional studies used the GRBASI scale. The GRBASI is a Japanese scale, based on ISSHIKI's work on hoarseness, used internationally. Initially conceived as GRBAS, in 1996 it had the addition of the instability factor I, by the authors Dejonckere, Remacle & Fresnel-Elbaz^(205)^. It is noteworthy the large presence of JPA scales created by the researchers themselves. The use of non-validated scales and instruments generates a difficulty in the comparison with other studies, in the clinical application and in the reliability of the findings, since they have no proven validity^(193)^, and the results cannot be reproduced.

The acoustic evaluation of the voice is one of the instrumental evaluations of the clinical speech therapist and appeared in 61 studies in the review. It is known that acoustic analysis quantifies the sound signal, making vocal analysis more objective. We reiterate that JPA is still the gold standard in vocal clinic and that acoustic analysis is complementary^(83)^.

In general, the acoustic analysis practiced in vocal clinic can be performed by extraction of acoustic parameters or by visual analysis of the spectrographic tracing. The review results show that traditional measures of automatic extraction of the fundamental frequency, noise measurements, and signal perturbations are more widely studied and published. This type of acoustic analysis, until recently, was what the Brazilian vocal clinic had as knowledge and available resource. However, currently, it is understood that in dysphonic individuals, this traditional analysis is not the best measure of evaluation, since the extraction of classical measures is performed in the time domain^(206)^. The signs of dysphonic voices are type 2 and 3, and do not produce reliable measurements^(197)^. In these cases, one can then opt for a descriptive analysis of the spectrographic tracing. Another option is to use multiparametric measurements that are more suitable for evaluations of dysphonic voices, offering more reliable and reliable results^(140)^.

ASHA recommends the Cepstral Peak Proeminence (CPP) for acoustic analysis of the dysphonic individual^(6)^. Only one study that used this measure was included in this review. Such data indicate the need for greater investment in these measures, as well as in other multiparametric acoustic measures in Brazil.

Few studies were found that performed laryngeal imaging exams. In Brazil, laryngeal imaging exams are not part of the phonoaudiological procedures. However, this is a necessary procedure for laryngeal diagnosis, a requirement to be able to classify the type of dysphonia. The instrument recommended by the American Academy of Otolaryngology-Head and Neck Surgery is the laryngostroboscopy^(207)^, in conjunction with a rigorous clinical history and complete medical evaluation^(208)^.

Aerodynamic evaluation was studied in 20 articles included in the review. It is a clinical instrumental evaluation that allows noninvasive measurements of glottal parameters that make up the vocal production^(195)^. Besides the measurements of maximum phonatory time, which help in the inference of physiological data such as glottal closure and tension during phonation, other aerodynamic measurements are hardly part of clinical vocal practice.

The most commonly used measure for aerodynamic assessment in the studies analyzed in this research was maximum phonation time, which is easy to obtain and non-invasive, widely used in clinical phonoaudiological practice^(209)^ to help describe vocal behavior. As for aerodynamic evaluation, ASHA recommends obtaining measurements of mean airflow rate and mean subglottic pressure. There is also an orientation to collect fundamental frequency measures and sound pressure measures, which are acoustic measures, collected simultaneously with the aerodynamic ones^(6,210)^.

With regard to intervention data, studies that measured immediate effects were more frequent. In longitudinal interventions, 6-session and 12-session interventions were more frequent. The average time of sessions most used by the researchers was 60 minutes, with a weekly frequency. It is observed that the frequency of 6-12 sessions may come from the influence of American publications, since 6 sessions correspond to the number of sessions covered by the American health insurance^(209)^.

In Brazil, the SBF (Brazilian Society of Speech Therapy) and CFF (the Federal Council of Speech Therapy) therapy beacons seek to guide the frequency, duration and amounts of voice therapy sessions for various conditions. The vocal alterations range from pediatric dysphonia to the rehabilitation of laryngectomized patients. There are recommendations for a number of sessions between eight and 24 sessions, 30 to 45 minutes each session, with weekly frequency of one to three times a week, varying according to the type of alteration and age of the patient^(2)^.

Of the studies on psychometric properties of instruments, only five were validation studies, the most frequent being translation and cross-cultural adaptation of instruments into Brazilian Portuguese. Erthal^(211)^ states that Psychometrics is a group of techniques that enables the quantification of psychic phenomena. Among the options for instrument validation we highlight the rules of the Scientific Advisory Committee of the Medical Outcomes Trust-SAC^(212)^, frequently used in national validation studies by the Classical Test Theory in the voice area. To be used, protocols need to be formally developed and psychometrically tested, thus ensuring evidence of validity, reliability, and fairness^(200)^. Translation and cross-cultural adaptation is an initial part of validation. However, only translation and cross-cultural adaptation is not sufficient for an instrument to be considered applicable and valid in a language^(213)^.

Regardless of the design, some studies did not report essential data so that their findings could be interpreted and their evidence useful for practice, which is an important opportunity to improve the description of the experiments. There were studies that did not report the age range, the sex of the subjects studied, and the time and frequency of the sessions. In addition, some studies used scales created by the researchers, which do not allow comparison of their findings. The most frequent assessment instruments in aerodynamic measures, in acoustic measures, and in self-assessment are not the instruments recommended by ASHA. However, it should be noted that the year of study selection and ASHA publication is close, and it will be necessary to review this information again in the future.

Therefore, it is necessary to reflect on the importance of a detailed and precise methodological description, as well as the standardization of procedures and measures for use in research. Evidence-based clinical practice is a necessity for the improvement of vocal clinic. However, it is necessary that some changes and standardization occur in research so that their findings are valid for clinical inference, and to be possible the implementation of evidence in practice.

## CONCLUSION

The results allow us to conclude that there was a gradual increase in the number of publications in the area of voice in the journal CoDAS. The procedures and characteristics of the publications were heterogeneous. Researchers in clinical and professional voice have a preference for more cross-sectional studies and with a sample of adults and of both sexes. Cross-sectional studies with self-assessment outcomes, experimental studies of immediate effect, and studies measuring psychometric properties that performed translation and cross-cultural adaptation were more frequent. There are uninformed data on relevant parameters for the applicability of the studies such as age range, sex, and temporal parameters of interventions, besides a high index of scales created by the researchers.
