# Supplementary material for: Mapping of the characteristics in the CoDAS journal publications in the voice area: a scoping review
Source: Codas. 2023 Jun 5;35(3):e20220088. doi: 10.1590/2317-1782/20232022088en (PMC10266801; doi:10.1590/2317-1782/20232022088en)
Supplement: Translation

# Translation: Mapeamento das características das publicações da revista CoDAS na área de voz: uma revisão de escopo

**DOI:** 10.1590/2317-1782/20232022088pt

**Authors:** Walter Williams Albrechet Chamun, Vanessa Veis Ribeiro, Glaucya Madazio, Mara Behlau

**Affiliations:** 1 Centro de Estudos da Voz - CEV - São Paulo (SP), Brasil.; 2 Departamento de Fonoaudiologia, Universidade Federal da Paraíba - UFPB - João Pessoa (PB), Brasil.; 3 Departamento de Fonoaudiologia, Universidade Federal de São Paulo - Unifesp - São Paulo (SP), Brasil.

**Keywords:** Voz, Desfechos, Procedimentos, Disfonia, Distúrbios da Voz

## Abstract

**Objetivo:**

Mapear e descrever as características das publicações da revista CoDAS na área da voz.

**Estratégia de pesquisa:**

Foi realizada uma busca eletrônica na base de dados Scielo pelo descritor voz.

**Critérios de seleção:**

Foram selecionados os estudos publicados na revista CoDAS, na área de voz.

**Análise dos dados:**

Foram extraídos dados específicos por delineamento. Os dados foram sintetizados por análise descritiva, e analisados de forma narrativa.

**Resultados:**

Foram mais frequentes estudos publicados em 2019, com delineamento transversal. Foram mais frequentes estudos com amostra na faixa etária de 18-59 anos. Nos estudos transversais, foi mais frequente a medida de resultado de autoavaliação vocal. Nos estudos de intervenção, a maioria foi de efeito imediato de uma sessão. Dentre os estudos de validação, foram mais frequentes os procedimentos de tradução e adaptação transcultural.

**Conclusão:**

Houve aumento gradual de estudos em voz, porém, as características das publicações foram heterogêneas.

## INTRODUÇÃO

No mundo contemporâneo da área da saúde, a busca por conhecimento científico tomou lugar de destaque, com a finalidade principal de fomentar a prática clínica. Nesse sentido, a produção da evidência por meio da publicação de um artigo científico é uma forma de transmitir à comunidade científica e clínica dados sobre o desenvolvimento de novos procedimentos, instrumentos e intervenções, além da análise de fatores de risco e realidade epidemiológica, nas diversas áreas da ciência^(1)^, assim como na Fonoaudiologia.

No Brasil, a voz é uma das áreas da Fonoaudiologia reconhecida como especialidade desde 2006^(2)^. A partir dessa resolução, a área de voz avançou ainda mais como especialidade reconhecida e em grande evolução.

A força e o reconhecimento de uma profissão ou área estão em grande parte fundamentadas na qualidade dos artigos publicados em revistas com revisão dos pares. A produção científica brasileira na área de voz é ampla, o que contribui e fortalece a prática.

Historicamente, as primeiras pesquisas na área de voz, assim como nas ciências da saúde e fonoaudiológicas de modo geral, abordavam delineamento de opiniões de especialistas e séries de casos da prática clínica. Tratava-se dos primeiros passos científicos de uma área jovem, porém, as conclusões desses estudos não podiam, ainda, ser generalizáveis. Já a prática baseava-se na aprendizagem adquirida durante a formação dos clínicos e as dúvidas destes profissionais eram tiradas diretamente com especialistas reconhecidos. No decorrer do tempo, as pesquisas foram se aprimorando, e o foco passou a ser a disponibilidade de evidências para aprimorar a prática clínica, bem como a implementação das evidências. Atualmente, o desafio do clínico vocal é a escolha da melhor evidência científica para embasar a sua prática^(3,4)^.

Tais mudanças decorreram, em grande parte, de um movimento denominado de prática baseada em evidências (PBE), que busca assegurar a qualidade dos cuidados dos profissionais da saúde, incluindo os fonoaudiólogos, visando uma melhor tomada de decisão frente a cada caso^(5).^ A PBE consiste em três etapas: 1) O clínico identifica um problema ou dúvida em sua prática; 2) O pesquisador transforma esse problema/dúvida em pergunta de pesquisa e desenvolve um estudo para respondê-la com base em evidências; 3) O clínico busca e seleciona o estudo, e aplica as evidências na sua prática clínica, a partir de uma análise crítica que leva em consideração sua experiência prática e as perspectivas do paciente^(6)^. Dessa forma, a execução da PBE não depende somente dos pesquisadores, mas principalmente dos clínicos que devem atuar como integradores da ciência e da clínica, avaliadores críticos de dados, e executores na aplicação das evidências em seus casos clínicos^(7)^. Pode-se dizer que o desenvolvimento contínuo da Fonoaudiologia e da área de voz está diretamente ligado à prática da PBE^(8)^, embora a PBE ainda não seja frequente na área de Voz.

O Brasil, atualmente, possui quatro revistas específicas da Fonoaudiologia que contribuem para a PBE, e uma delas é a CoDAS. O nome da revista CoDAS *(Communication Disorders, Audiology and Swallowing)*, sigla em inglês para Distúrbios da Comunicação, Audiologia e Deglutição, foi criado para ser curto e fácil de ser lembrado, e considerou as áreas principais da Fonoaudiologia.

A revista CoDAS é um periódico que vem se adaptando às necessidades da academia e da clínica fonoaudiológica. Inicialmente, foi denominada Pró-Fono Revista de Atualização Científica (2005-2010). Em 2010, passou a se chamar Jornal da Sociedade Brasileira de Fonoaudiologia (2010-2012). Os artigos publicados a partir de 2010 encontram-se disponíveis na plataforma Scielo com acesso aberto. Em sua última reestruturação em 2013, passou a ser chamada de CoDAS e com editorial centralizado como publicação única da Sociedade Brasileira de Fonoaudiologia. A revista possui como fontes de indexação a *Web of Science*, MEDLINE/PubMed, Scopus, PsycINFO, *Scientific Electronic Library Online* (SciELO), *Linguistics and Language Behavior Abstracts* (CSA), Literatura Latino-Americana e do Caribe em Ciências da Saúde (LILACS), *Sociedad Iberoamericana de Información Científica* (SIIC Data Bases), e o *Directory of Open Access Journals* (DOAJ).

Considerando-se a importância do periódico para a pesquisa e a clínica na área de voz, torna-se relevante mapear as publicações da área nela realizadas. Tais dados fornecerão um panorama geral sobre o escopo das publicações no que se refere aos delineamentos, amostras, desfechos e prescrição de exercícios. Acredita-se que tais dados contribuirão para identificar o que vem sendo realizado no país, mapear os avanços, apontar as limitações e necessidades da área e auxiliar o fonoaudiólogo na busca da evidência em prol de um objetivo final que é fornecer o melhor atendimento para os pacientes da clínica vocal. Além disso, o mapeamento realizado por uma revisão de escopo pode contribuir para direcionamentos futuros na área e crescimento da ciência.

Dessa forma, o objetivo geral desta pesquisa foi mapear e descrever as características das publicações da revista CoDAS na área de voz.

## ESTRATÉGIA DE PESQUISA

O presente estudo tem delineamento de revisão de escopo e seguiu as recomendações da *Joanna Briggs Institute Manual for Evidence Synthesis for Scoping Reviews* ^(9)^ e do PRISMA-ScR^(10)^. O protocolo da presente revisão de escopo foi registrado na Open Science Framework (doi:10.17605/OSF.IO/VFWN7).

Para elaborar a pergunta de pesquisa foi utilizada a sigla PCC: População - indivíduos disfônicos e não disfônicos; Conceito - características amostrais e metodológicas; Contexto - revista CoDAS. Dessa forma, a pergunta de pesquisa que subsidiou seu desenvolvimento foi: Quais as principais características amostrais e metodológicas dos estudos com indivíduos disfônicos e não disfônicos publicados na revista CoDAS?

A busca foi realizada na base de dados Scielo, utilizando-se o descritor “voz”. Foram aplicados os filtros de periódico selecionando-se apenas a CoDAS, e de período de publicação até dezembro de 2019. A busca foi realizada no mês de agosto de 2020.

## CRITÉRIOS DE SELEÇÂO

Os critérios de inclusão utilizados para considerar os estudos para essa revisão foram: artigos publicados no periódico CoDAS (Pró-Fono Revista de Atualização Científica, Jornal da Sociedade Brasileira de Fonoaudiologia e CoDAS), período até dezembro de 2019, na área de voz, com população de indivíduos disfônicos e não disfônicos. Em junho de 2022 o artigo foi atualizado com as publicações de janeiro de 2020 a dezembro de 2021. Foram excluídos os estudos interdisciplinares em que o foco não era a área de voz (desfechos de outras áreas), e os estudos secundários (revisão de literatura).

Os procedimentos utilizados para selecionar os estudos e aplicar os critérios de elegibilidade foram: leitura do título; leitura do resumo e leitura na íntegra dos artigos. A seleção foi realizada pelo autor principal entre agosto e novembro de 2020.

## ANÁLISE DE DADOS

A extração e análise dos dados foi realizada por dois autores. A fim de facilitar a extração e análise dos dados, os estudos selecionados foram separados em três grupos, de acordo com o delineamento: observacional^(11)^; intervenção^(11)^; e, validação de instrumentos. Os dados extraídos dos estudos foram:

1. Observacional (Transversal, Coorte e Caso-controle): autores, ano, país, instituição, delineamento, área, faixa etária, sexo, cálculo amostral, desfechos, autoavaliação, avaliação perceptivo-auditiva, análise acústica, aerodinâmica;
2. Intervenção (Quasi-experimental, Experimental e Intervenção antes e após): autores, ano, país, instituição, delineamento, área, faixa etária, sexo, cálculo amostral, número de sessões, tempo de sessão, frequência de sessão;
3. Tradução, adaptação transcultural e validação de instrumentos: autores, ano, país, instituição, delineamento, área, faixa etária, sexo, cálculo amostral, tradução e adaptação transcultural.

A síntese dos dados foi apresentada de forma descritiva por meio de tabelas e gráficos com análise de frequência. A análise de dados foi realizada no formato narrativo.

## RESULTADOS

A Figura 1 mostra que 2742 estudos foram identificados na base de dados Scielo. Durante a primeira fase de seleção 2530 estudos foram excluídos por não serem da área de Voz ou por serem estudos interdisciplinares sem desfechos na área de voz, perfazendo assim 212 estudos. Durante a segunda fase de seleção, 36 foram excluídos com base no delineamento (estudos secundários). Desta forma, foram selecionados 176 estudos^(12-187)^.

**Figura 1:** Fluxograma de busca e seleção

Legenda: n = frequência absoluta

As publicações na área de voz foram mais frequentes nos anos de 2019 (n=20; 11,4%) e 2013 (n=19; 10,8%), como indica a Figura 2.

**Figura 2:** Análise descritiva da frequência de artigos da área de voz por ano de publicação na CoDAS

A Tabela 1 mostra que, na revista CoDAS, os artigos desenvolvidos no Brasil foram mais frequentes (n=165; 93,8%), seguidos pelos Estados Unidos da América (n=3; 1,7%). As instituições que mais publicaram foram Centro de Estudos da Voz (n=46; 26,1%), Universidade de São Paulo (n=20; 11,4%), e Universidade Federal de São Paulo (n=17; 9,7%), empatada com estudos multicêntricos (n=17; 9,7%).

**Tabela 1:** Características dos estudos

Variável e categorias	n	%
**País**		
Brasil	165	93,8
Chile	3	1,7
Estados Unidos	3	1,7
Bélgica	1	0,6
Brasil e Bélgica	1	0,6
Brasil e Canadá	1	0,6
Holanda	1	0,6
Itália	1	0,6
**Instituição**		
Centro de Estudos da Voz - São Paulo / SP	46	26,1
Universidade de São Paulo - São Paulo / SP	20	11,4
Multicêntricas	17	9,7
Universidade Federal de Minas Gerais - Belo Horizonte / MG	17	9,7
Universidade Federal de São Paulo - São Paulo / SP	16	9,1
Universidade Federal da Paraíba - João Pessoa / PB	10	5,7
Pontifícia Universidade Católica de São Paulo - São Paulo / SP	7	4,0
Universidade Estadual Paulista - Marília / SP	5	2,8
Universidade Federal de Santa Catarina - Florianópolis / SC	4	2,3
Universidade Federal do Espírito Santo - Vitória / ES	4	2,3
Universidade Federal de Santa Maria / RS	3	1,7
Santa Casa de Misericórdia de São Paulo - São Paulo / SP	3	1,7
Universidade Metodista de Piracicaba - Piracicaba / SP	3	1,7
Universidade Estadual do Centro-Oeste-Irati / PR	2	1,1
Universidade Federal da Sergipe - Lagarto / SE	2	1,1
Columbia University - New York (USA)	1	0,6
Hospital AG Sint-Jan - Bruges (Belgium)	1	0,6
Pontificia Universidade Católica de Goiás - Goiania / GO	1	0,6
Santa Casa de Misericórdia de Porto Alegre - ISCMPA - Porto Alegre / RS	1	0,6
Santa Casa de Misericórdia do Rio de Janeiro - Rio de Janeiro / RJ	1	0,6
Southern IIIinois University - Carbondale / IL (USA)	1	0,6
The Touro College and University System - New York (USA)	1	0,6
Universidad de Valparaiso - Valparaiso / Chile	1	0,6
Universidade Estadual de Campinas - Campinas / SP	1	0,6
Universidade Estadual de Montes Claros - Montes Claros/MG	1	0,6
Universidade Federal da Bahia - Salvador / BA	1	0,6
Universidade Federal de Ciências da Saúde de Porto Alegre - Porto Alegre / RS	1	0,6
Universidade Federal de Pernambuco - Recife/PE	1	0,6
Universidade Federal do Rio Grande do Sul - Porto Alegre / RS	1	0,6
Universidade Tuiuti do Paraná-UTP-Curitiba / PR	1	0,6
Universidade Veiga de Almeida e Ambulatório de Urologia Reconstrutura Universitário Pedro Ernesto - HUPE - Rio de Janeiro / RJ	1	0,6
Zuyd University of Applied Sciences - Heerlen (Holanda)	1	0,6
**Delineamento**		
Transversal	115	65,3
Quasi-experimental	27	15,3
Estudos de tradução, adaptação transcultural ou validação	24	13,6
Experimental	6	3,4
Intervenção antes e após	4	2,3

Legenda: n = frequência absoluta; % = frequência relativa percentual

A maioria dos estudos apresentou delineamento transversal (n=115; 59,7%), seguido de quasi-experimental (n=27; 15,3%), estudos de tradução, adaptação transcultural ou validação (n=24; 13,6%), experimental (n=6; 3,4%) e intervenção antes e após (n=4; 2,3%).

De acordo com a tabela 2, houve maior frequência de estudos de voz clínica (n=105; 59,7%). Foram mais frequentes estudos com amostra na faixa etária de 18-59 anos (n=96; 54,5%), e de ambos os sexos (n=102; 58,0%).

**Tabela 2:** Características da área e amostra dos estudos

Variável e categorias	N	%
**Área**		
Voz Clínica	105	59,7
Voz Profissional	71	40,3
**Faixa etária**		
18-59	96	54,5
60-103	42	23,9
0-18	21	11,9
Não informado	17	9,7
**Sexo**		
Ambos os sexos	102	58,04
Feminino	48	26,57
Não informado	17	9,09
Masculino	9	6,29

Legenda: n = frequência absoluta; % = frequência relativa percentual

Com relação aos 115 estudos transversais, a Tabela 3 mostra que os desfechos mais frequentes foram de autoavaliação vocal (n=102; 88,7%), o julgamento perceptivo-auditivo da voz - JPA (n=92; 80%) e a análise acústica da voz (n=61; 53%).

**Tabela 3:** Características dos estudos transversais (n=115)

Variável e categorias	n	%
**Desfechos (n=115)**		
Autoavaliação	102	88,7
JPA	92	80,0
Análise acústica	61	53,0
Avaliações de outras áreas	42	36,5
Imagem laríngea	22	19,1
Aerodinâmica	20	17,4
Outras avaliações vocais	10	8,7
**Autoavaliação (n=102)**		
ESV	14	13,7
QVV	10	9,8
IDV-10	7	6,9
Autoavaliação elaborada pelo pesquisador	6	5,9
IDV	6	5,9
IFV	6	5,9
CPV-P	5	4,9
EDTV	5	4,9
ITDV	5	4,9
QSHV	5	4,9
PPAV	4	3,9
URICA-VOZ	4	3,9
IDCM	4	3,9
PRRD	3	2,9
LSSV	2	2,0
SSPS	2	2,0
VcD	2	2,0
BORG CR10-BR	1	1,0
EAFV	1	1,0
IDCC	1	1,0
IDV-C	1	1,0
Lista de Sinais e Sintomas Vocais	1	1,0
LSU-BR	1	1,0
PDA	1	1,0
PEED	1	1,0
Quantidade de fala e intensidade de voz	1	1,0
QVV-P	1	1,0
RAVI	1	1,0
TVQ	1	1,0
**JPA (n=92)**		
Escalas criadas pelos pesquisadores	26	28,3
GRBASI	28	30,4
EAV-escala analógico visual	21	22,8
CAPE-V	12	13,0
EN (Escala Numérica)	1	1,1
GGDV (grau global de desvio da voz)	1	1,1
GT (grau de tensão)	1	1,1
VPAS	1	1,1
Likert (escala)	1	1,1
**Análise acústica (n=61)**		
Análise acústica tradicional	52	85,2
DDF (diagrama de desvio fonatório)	5	8,2
DDC (diadococinesia)	2	3,3
AVQI	1	1,6
CPPs	1	1,6
**Aerodinâmica (n=20)**		
TMF (tempo máximo fonatório)	19	95,0
Nasometria	1	5,0

Legenda: n = frequência absoluta; % = frequência relativa percentual; JPA = Julgamento Perceptivo auditivo; ESV = Escala de Sintomas Vocais; QVV = Qualidade de vida em Voz; IDV-10 = Índice de Desvantagem Vocal; IDV = Índice de Desvantagem Vocal; IFV = Índice de Fadiga Vocal; CVP-P = Condição de Produção Vocal do Professor; EDTV = Escala de Desconforto do Trato Vocal; ITDV = Índice de Triagem para o Distúrbio da Voz; QSHV = Questionário de Saúde e Higiene Vocal; PPAV = Protocolo de Perfil de Participação e Atividades Vocais; IDCM = Índice de Desvantagem para o Canto Moderno; PRRD = Protocolo de Rastreio do Risco de Disfonia; LSSV = Lista de Sinais e Sintomas Vocais; SSPS = Escala para Autoavaliação ao Falar em Público; VcD = Questionário Vivendo com Disartria; BORG CR10-BR = *Adapted Borg CR10 For Vocal Effort Ratings*; EAFV = Escala de Autopercepção de Fadiga Vocal; IDCC = Índice de Desvantagem Vocal no Canto Clássico; IDVC = Índice de Desvantagem para o Canto; LSU-BR= *The Levels of Speech Usage*; PDA = Protocolo de Disfunção Autônoma; PEED = Protocolo de Estratégia de Enfrentamento das Disfonias; QVV-P = Qualidade de Vida em Voz-Pediátrico; RAVI = Rastreio de Alterações Vocais no Idoso; TVQ = *Transgender Voice Questionnaire for Male to Female Transsexuals*; GRBASI = Escala Japonesa para avaliação de parâmetros perceptivos auditivos; EAV = Escala Analógico Visual; CAPE-V = Consenso da Avaliação Perceptivo Auditiva da Voz; EN = Escala Numérica; GGDV = Grau Global de Desvio da Voz; GT = Grau de Tensão; VPAS = Protocolo de Análise do Perfil Vocal de John Laver; LIKERT = Escala de autorrelato; DDF = Diagrama de Desvio Fonatório; DDC = Avaliação de Diadococinesia; AVQI = Acoustic Voice Quality Index; CPPS = *Smoothed Cepstral Peak Prominence*; TMF = Tempos Máximos Fonatórios; Nasometria = Avaliação de Nasalância

Nos estudos que analisaram o impacto autorreferido de um problema de voz, ou seja, a autoavaliação vocal (n=102), os instrumentos mais utilizados foram a Escala de Sintomas Vocais - ESV (n=14; 13,7%), Qualidade de Vida em Voz (QVV) (n=10; 9,8%) e o Índice de Desvantagem Vocal - IDV-10 (n=7; 6,9%). Já nos estudos que realizaram JPA (n=78), a escala GRBASI (n=28; 30,4%) e as escalas criadas pelos próprios pesquisadores (n=26; 28,3%) foram mais frequentemente usadas. Nos 61 estudos com análise acústica, a extração de parâmetros acústicos tradicionais (n=52; 85,2%) e o diagrama de desvio fonatório (DDF) (n=5; 8,2%) foram os mais publicados. Por fim, os poucos trabalhos que fizeram a análise aerodinâmica (n=20) estudaram o tempo máximo fonatório - TMF (n=19; 95%) e a nasometria (n=1; 5%).

Observou-se que dos 37 estudos de intervenção, 16 (43,2%) estudaram os efeitos imediatos, seguidos pelos que fizeram seis (n=3; 8,1%) ou doze sessões de intervenção (n=3; 8,1%).

O tempo de sessão mais frequente dentre os que relataram a informação foi de 60 minutos (n=6; 16,2%), sendo predominante a ausência dessa informação (n=10; 27%). Estudos cuja frequência de sessão foi única (n=16; 43,2%) foram os de maior ocorrência, seguidos da frequência semanal (n=9; 24,3%), conforme mostra a Tabela 4.

**Tabela 4:** Características dos estudos de intervenção (n=37)

Variável e categorias	n	%
**Número de sessões**		
1 (efeito imediato)	16	43,2
6	3	8,1
12	3	8,1
2	2	5,4
5	2	5,4
16	2	5,4
3	1	2,7
4	1	2,7
7	1	2,7
8	1	2,7
10	1	2,7
45	1	2,7
Não se aplica	3	8,1
**Tempo de sessão**		
Não informado	10	27,0
60 minutos	6	16,2
20 minutos	3	8,1
1 minuto	3	8,1
Não se aplica	3	8,1
30 minutos	2	5,4
3 minutos	1	2,7
5 minutos	1	2,7
10 minutos	1	2,7
15 minutos	1	2,7
16 minutos	1	2,7
40 minutos	1	2,7
45 minutos	1	2,7
64 minutos	1	2,7
90 minutos	1	2,7
1 sessão de 240 minutos / 4 sessões de 120 minutos	1	2,7
**Frequência de sessão**		
Única (efeito imediato)	16	43,2
1x semana	9	24,3
2x semana	3	8,1
Não informado	3	8,1
2 a 3x semana	1	2,7
4x semana	1	2,7
1x mês	1	2,7
Não se aplica	3	8,1

Legenda: n = frequência absoluta; % = frequência relativa percentual

A Tabela 5 mostra que dos 24 estudos de elaboração, adaptação e validação, 17 (70,8%) realizaram a fase de tradução e adaptação transcultural de instrumentos; 5 (20,8%) realizaram validação; e, apenas 2 (8,3%) elaboraram um novo instrumento.

**Tabela 5:** Características dos estudos de tradução, adaptação transcultural e validação (n=24)

Variável e categorias	n	%
Tradução e adaptação transcultural	17	70,8
Validação	5	20,8
Elaboração de instrumento	2	8,3

Legenda: n = frequência absoluta; % = frequência relativa percentual

## DISCUSSÃO

A pesquisa científica em Fonoaudiologia busca fornecer evidências confiáveis para a clínica^(159,163,185-187)^ a partir da prática baseada em evidências^(188).^ Nessa prática, os clínicos deparam-se com diferentes resultados, e precisam comparar evidências que respondam às suas perguntas e auxiliem na tomada de decisão em busca dos melhores resultados. Porém, essa comparação é dificultada quando se analisa pesquisas com diferentes procedimentos ou com diversos instrumentos usados para uma mesma finalidade. A revista CoDAS ocupa um lugar de destaque no cenário nacional de publicações de pesquisas científicas. Nesse sentido, mapear procedimentos e analisar suas frequências mostram tendências, limitações, e podem contribuir para a prática clínica, bem como para o desenvolvimento de outros estudos.

Os resultados da presente revisão de escopo mostraram que houve uma curva ascendente não linear das publicações na revista CODAS nos últimos anos analisados, com uma ascensão nos anos de 2013 e 2020. O maior número de publicações no ano de 2013 pode ser explicado pelas mudanças de estratégias editoriais, como o novo nome da revista que passou a ser denominada CoDAS, a inserção de editores de área, a maior participação de colegas estrangeiros e a profissionalização da secretaria editorial. Além disso, o ano de 2013 apresentou uma melhoria na definição dos objetivos dos estudos brasileiros nos desenhos dos experimentos, assim como a ampliação de trabalhos multicêntricos^(189)^. Quanto ao ano de 2019, a revista foi marcada pela melhoraria da indexação e internacionalização do periódico.

As publicações nacionais são as mais frequentes na revista CoDAS. Dentre os 11 artigos internacionais, os Estados Unidos da América e o Chile publicaram três, mas vale lembrar que apenas recentemente a revista passou a ter visibilidade internacional com o inglês como idioma obrigatório. Os artigos internacionais são tanto publicados em conjunto entre instituições nacionais e estrangeiras, como de autoria apenas estrangeira. Acredita-se que, mesmo diante de uma revista ainda forte e reconhecida nas publicações da Fonoaudiologia brasileira, o aumento da internacionalização, como a recente indexação da revista na *Web of Science*, trará maior visibilidade internacional e alcance, além de mais dados de crescimento e pesquisas para a revista.

O Centro de Estudos da Voz - CEV foi a instituição de ensino que mais publicou na CoDAs na área da voz, seguida pela Universidade de São Paulo - USP, Universidade Federal de São Paulo - UNIFESP e pelas publicações multicêntricas. O CEV é uma instituição de ensino e pesquisa na área de comunicação humana que completou 40 anos em 2021, que oferece um Curso de Especialização em Voz - CECEV, cuja primeira turma formada foi em 1993 (A voz do professor, Sinpro/CEV). O CECEV, em 2021, montou sua 24ª turma e todos os alunos são incentivados e orientados a desenvolver monografia de conclusão de curso para apresentação em congressos e publicação. A USP é uma universidade pública tradicional na área, que possui três campus com o curso de Fonoaudiologia, em São Paulo, em Ribeirão Preto, e em Bauru. A UNIFESP é uma das universidades mais antigas no ensino e na pesquisa, e é reconhecida também pelo esforço científico na área da voz. Trata-se de uma universidade pública que tem o objetivo de desenvolver atividades inter-relacionadas de ensino, pesquisa e extensão, com ênfase no campo específico das ciências da saúde^(190)^. A UNIFESP conta com programas de iniciação científica ainda na graduação e os programas de aperfeiçoamento e especialização estão vinculados à produção científica e publicação. Na sequência, os resultados apontaram para as publicações multicêntricas. Trata-se de uma tendência mundial e um bom indicativo para a revista, que mostra que os pesquisadores de institutos e universidades vem se unindo em prol da melhoria do desenvolvimento científico. A ampliação de trabalhos multicêntricos pode oferecer uma representação geográfica nacional mais abrangente, além deles serem fundamentais para o desenvolvimento de grandes projetos.

Os estudos observacionais do tipo transversais, que são delineamentos simples, rápidos e de mais fácil execução foram mais comumente encontrados na revisão. São delineamentos de pesquisa tidos como excelente método para descrever características de uma população. Contudo, são válidos apenas para aquele determinado local e momento e, com o passar do tempo, seus achados já não podem ser usados para a clínica^(191,192)^. É importante ressaltar que o delineamento está diretamente relacionado à pergunta clínica que se pretende responder, sendo que esse tipo de estudo é mais indicado para descrição de características, acurácia diagnóstica e prevalência de doenças^(3)^.

Os estudos transversais foram seguidos pelos quasi-experimentais, sendo o mais tipo mais frequente dentre os estudos de intervenção. Estudos quasi-experimentais são intervenções controladas, mas que não possuem randomização da amostra. A falta de randomização gera risco de viés de seleção. Já os experimentais, também denominados de ensaios clínicos randomizados, são considerados estudos de alto nível de evidência^(3)^ e padrão-ouro para análise de eficácia de intervenções^(192)^, porém, foram observadas apenas seis publicações com esse delineamento^.^ Apesar da qualidade das publicações ter aumentado no último decênio, estando ainda no processo de crescimento e modificações estruturais, poucas pesquisas publicadas em revistas especializadas incorporaram a metodologia adequada e respondem às perguntas clínicas satisfatoriamente, tanto nas revistas brasileiras quanto nas publicações internacionais^(193)^.

Observou-se um equilíbrio entre os estudos de voz clínica e de voz profissional, porém, ainda com maior frequência na área de voz clínica, historicamente mais presente na área de Voz. É importante ressaltar que ambas as subáreas vêm sendo contempladas nas publicações da revista CoDAS, ampliando as possibilidades de busca de evidência para a prática da clínica vocal, no treinamento da voz profissional e na terapia da voz clínica.

A maioria dos estudos incluiu participantes adultos (18-59 anos), seguidos pelos idosos (60-103 anos) e pelas crianças e adolescentes (0-18 anos). Os adultos também representam a maioria dos pacientes que buscam a clínica vocal^(194)^.A população de idosos vem ganhando espaço não apenas na clínica vocal, mas em todos os estudos da área da saúde, pelas mudanças demográficas e pela busca de longevidade vocal e qualidade de vida, apesar de ainda serem poucos em comparação aos adultos^(195,196)^. As crianças são minoria na procura de atendimento clínico, apesar dos dados de prevalência da disfonia infantil ser de até 38% da população pediátrica^(151)^. Acredita-se que a disfonia pediátrica apresenta menos estudos pela necessidade de autorização dos pais que não necessariamente querem seus filhos como sujeito de pesquisa^(151)^. A ocorrência de estudos com crianças encontrada nessa revisão vai ao encontro da literatura^(151,196)^. Cabe ressaltar aqui que algumas pesquisas não informaram a faixa etária dos participantes, embora sejam dados essenciais para a aplicabilidade e interpretação dos resultados.

A maior parte das pesquisas contemplou ambos os sexos em suas análises. Esse dado é positivo e relevante porque as diferenças anatomofisiológicas existentes entre os sexos levam à necessidade de estudos específicos e interpretações diversas, além dos valores de normalidade diferentes para algumas avaliações^(197-199)^.

Ao considerar apenas os estudos transversais, observa-se que o desfecho mais frequente foi a autoavaliação, seguido do JPA. Essas duas avaliações fazem parte das avaliações não-instrumentais que compõe a avaliação multidimensional da voz^(6)^. A autoavaliação traz dados que não podem ser obtidos na avaliação clínica realizada pelo fonoaudiólogo e servem para quantificar a percepção do sujeito acerca da influência da sua voz nas diferentes atividades diárias^(200)^. O bem-estar total não contempla apenas a ausência da doença, mas também a autopercepção do indivíduo em sua condição e os impactos em diversos aspectos da sua vida^(201-203)^.

A revisão mostrou que os instrumentos de autoavaliação mais frequentemente utilizados foram a Escala de Sintomas Vocais (ESV), seguido pelo Qualidade de Vida em Voz (QVV) e pelo Índice de Desvantagem Vocal (IDV-10). Estes instrumentos, todos validados para o português brasileiro^(23,37,204)^ são amplamente utilizados no dia a dia da clínica vocal por serem rápidos, de fácil aplicação e ainda por apresentarem propriedades psicométricas confiáveis, capazes de classificar indivíduos com e sem disfonia. A ESV é um dos instrumentos mais robustos na área da voz por suas propriedades psicométricas e busca analisar a autopercepção de sintomas vocais^(37)^. O IDV-10, versão reduzida do IDV-30, é um instrumento rápido, prático e confiável para mensurar a desvantagem vocal em indivíduos com problemas de voz^(68).^ O QVV foi o primeiro instrumento de autoavaliação vocal a ser validado para o português brasileiro, muito aplicado na clínica vocal, que permite analisar o impacto de um problema de voz na qualidade de vida de indivíduos disfônicos^(204)^ No atendimento de um paciente disfônico, a *American Speech-Language-Hearing Association* (ASHA) recomenda o uso de protocolos de autoavaliação, mas não cita um específico^(6)^ provavelmente porque as propriedades psicométricas dos instrumentos são diferentes entre as validações de cada língua e porque os instrumentos possuem diferentes objetivos.

O JPA é considerado padrão-ouro na avaliação vocal, capaz de qualificar a qualidade da voz e quantificar o grau de desvio^(205)^. A maioria dos estudos transversais utilizou a escala GRBASI. A GRBASI é uma escala japonesa, baseada nos trabalhos de ISSHIKI sobre rouquidão, usada internacionalmente. Inicialmente concebida como GRBAS, em 1996 teve o acréscimo do fator instabilidade I, pelos autores Dejonckere, Remacle & Fresnel-Elbaz^(205)^. Ressalta-se a grande presença de escalas de JPA criadas pelos próprios pesquisadores. O uso de escalas e instrumentos não validados gera uma dificuldade na comparação com outros estudos, na aplicação clínica e na confiabilidade dos achados, visto que não tem validade comprovada^(193)^, e os resultados não podem ser reproduzidos.

A avaliação acústica da voz é uma das avaliações instrumentais do fonoaudiólogo clínico vocal e apareceu em 61 estudos da revisão. Sabe-se que a análise acústica quantifica o sinal sonoro, tornando mais objetiva a análise vocal. Reiteramos que o JPA ainda é padrão-ouro na clínica vocal e que a análise acústica é complementar^(83)^.

De forma geral, a análise acústica praticada na clínica vocal pode ser realizada por meio da extração de parâmetros acústicos ou por análise visual do traçado espectrográfico. Os resultados da revisão demonstram que as medidas tradicionais de extração automática da frequência fundamental, medidas de ruído e perturbações do sinal são mais estudadas e publicadas. Este tipo de análise acústica, até há pouco tempo, era o que a clínica vocal brasileira tinha como conhecimento e recurso disponível. Contudo, atualmente, entende-se que, em indivíduos disfônicos, esta análise tradicional não é a melhor medida de avaliação, visto que a extração das medidas clássicas é realizada no domínio do tempo^(206)^. Os sinais de vozes disfônicas são do tipo 2 e 3, e não produzem mensurações confiáveis^(197)^. Nestes casos, pode-se, então, optar por uma análise descritiva do traçado espectrográfico. Outra opção é utilizar medidas multiparamétricas que são mais indicadas para avaliações de vozes disfônicas, oferecendo resultados mais fidedignos e confiáveis^(140)^.

A ASHA recomenda o *Cepstral Peak Proeminence* (CPP) para a análise acústica do indivíduo disfônico^(6)^. Apenas um estudo que utilizou essa medida foi incluído na presente revisão. Tais dados indicam a necessidade de maiores investimentos nessas medidas, bem como em outras medidas acústicas multiparamétricas no Brasil.

Foram encontrados poucos estudos que fizeram exames de imagem da laringe. No Brasil, os exames de imagem de laringe não fazem parte dos procedimentos fonoaudiológicos. Contudo, trata-se de um procedimento necessário para o diagnóstico laríngeo, requisito para que seja possível classificar o tipo de disfonia. O instrumento recomendado pela Academia de Laringologia e Cirurgia de Cabeça e Pescoço dos Estados Unidos para a avaliação laríngea é a laringoestroboscopia^(207)^, em conjunto com um histórico clínico rigoroso e a avaliação médica completa^(208)^.

A avaliação aerodinâmica foi estudada em 20 artigos incluídos na revisão. Ela é uma avaliação clínica instrumental que permite obter medidas não invasivas de parâmetros glóticos que compõem a produção vocal^(195)^. Além das medidas de tempo máximo fonatório, que auxiliam na inferência de dados fisiológicos como fechamento glótico e tensão à fonação, outras medidas aerodinâmicas dificilmente fazem parte da prática da clínica vocal.

A medida mais utilizada para avaliação aerodinâmica nos estudos analisados nesta pesquisa foi de tempo máximo de fonação, de fácil obtenção e não invasiva, muito utilizada na prática clínica fonoaudiológica^(209)^ para auxiliar na descrição do comportamento vocal. Com relação a avaliação aerodinâmica, a ASHA orienta a obtenção das medidas de taxa média de fluxo de ar e pressão subglótica média. Há, também, uma orientação de coleta de medidas de frequência fundamental e medidas de pressão sonora, que são medidas acústicas, coletadas simultaneamente as aerodinâmicas^(6,210)^.

Com relação aos dados de intervenção, foram mais frequentes os estudos que mensuraram efeitos imediatos. Nas intervenções longitudinais, foram mais frequentes as de 6 sessões e 12 sessões. O tempo médio de sessões mais utilizado pelos pesquisadores foi de 60 minutos, com frequência semanal. Observa-se que a frequência de 6-12 sessões pode advir da influência das publicações americanas, pelo fato de que 6 sessões correspondem ao número de sessões cobertas pelo seguro saúde americano^(209)^.

No Brasil, os balizadores de terapia da SBFa (Sociedade Brasileira de fonoaudiologia) e do CFF (Conselho federal de Fonoaudiologia) procuram orientar a frequência, duração e quantidades de sessões de terapia de voz para diversos quadros. As alterações vocais vão desde disfonia pediátrica até a reabilitação de pacientes laringectomizados. Há recomendações de um número de sessões entre oito e 24 sessões, 30 a 45 minutos cada sessão, com frequência semanal de uma a três vezes na semana, variando em função do tipo de alteração e idade do paciente^(2)^.

Dos estudos de propriedades psicométricas de instrumentos, apenas cinco foram de validação, sendo mais frequente a de tradução e adaptação transcultural de instrumentos para o português brasileiro. Erthal^(211)^ afirma que a Psicometria é um grupo de técnicas que viabiliza a quantificação de fenômenos psíquicos. Dentre as opções para a validação de instrumentos destacamos as regras do *Scientific Advisory Committee of the Medical Outcomes Trust-SAC* ^(212)^, frequentemente utilizadas nos estudos nacionais de validação por Teoria Clássica dos Testes na área de voz. Para serem utilizados os protocolos precisam ser formalmente desenvolvidos e psicometricamente testados, garantindo assim evidências de validade, confiabilidade e equidade^(200)^. A tradução e adaptação transcultural é uma parte inicial da validação. Porém, apenas a tradução e adaptação transcultural não é suficiente para que um instrumento seja considerado aplicável e válido em uma língua^(213)^.

Independentemente do delineamento, alguns estudos não informaram dados essenciais para que seus achados possam ser interpretados e suas evidências úteis para a prática, sendo essa uma oportunidade importante para a melhoria na descrição dos experimentos. Foram encontrados estudos que não informaram a faixa etária, o sexo dos sujeitos pesquisados, e o tempo e frequência de sessão. Além disso, houve estudos utilizaram escalas criadas pelos pesquisadores, que não permitem comparação dos seus achados. Os instrumentos mais frequentes de avaliação em medidas aerodinâmicas, em medidas acústicas e de autoavaliação não são os instrumentos recomendados pela ASHA. Porém, cabe ressaltar que o ano de seleção dos estudos e de publicação da ASHA é próximo, sendo necessário que futuramente essa informação seja novamente revisada.

Diante disso, faz-se necessária uma reflexão sobre a importância de uma descrição metodológica detalhada e precisa, bem como da padronização de procedimentos e medidas para o uso na pesquisa. A prática clínica baseada em evidências é uma necessidade para o aprimoramento da clínica vocal. Porém, é necessário que algumas mudanças e padronizações ocorram nas pesquisas para que seus achados sejam válidos para a inferência clínica, e para que seja possível a implementação da evidência na prática.

## CONCLUSÃO

Os resultados permitem concluir que houve aumento gradual no número de publicações na área de voz da revista CoDAS. Os procedimentos e características das publicações foram heterogêneos. Os pesquisadores em voz clínica e profissional têm por preferência mais estudos transversais e com uma amostra de adultos e de ambos os sexos. Foram mais frequentes os estudos transversais com desfechos de autoavaliação, estudos experimentais de efeito imediato, e estudos de mensuração de propriedades psicométricas que realizaram tradução e adaptação transcultural. Há dados não informados de parâmetros relevantes para a aplicabilidade dos estudos como faixa etária, sexo e parâmetros temporais de intervenções, além de um alto índice de escalas criadas pelos pesquisadores.
